# Pancreatic Cancer Prediction Through an Artificial Neural Network

**DOI:** 10.3389/frai.2019.00002

**Published:** 2019-05-03

**Authors:** Wazir Muhammad, Gregory R. Hart, Bradley Nartowt, James J. Farrell, Kimberly Johung, Ying Liang, Jun Deng

**Affiliations:** ^1^Department of Therapeutic Radiology, School of Medicine, Yale University, New Haven, CT, United States; ^2^Department of Internal Medicine, School of Medicine, Yale University, New Haven, CT, United States

**Keywords:** pancreatic cancer, cancer risk, cancer prediction, artificial neural network, big data

## Abstract

Early detection of pancreatic cancer is challenging because cancer-specific symptoms occur only at an advanced stage, and a reliable screening tool to identify high-risk patients is lacking. To address this challenge, an artificial neural network (ANN) was developed, trained, and tested using the health data of 800,114 respondents captured in the National Health Interview Survey (NHIS) and Pancreatic, Lung, Colorectal, and Ovarian cancer (PLCO) datasets, together containing 898 patients diagnosed with pancreatic cancer. Prediction of pancreatic cancer risk was assessed at an individual level by incorporating 18 features into the neural network. The established ANN model achieved a sensitivity of 87.3 and 80.7%, a specificity of 80.8 and 80.7%, and an area under the receiver operating characteristic curve of 0.86 and 0.85 for the training and testing cohorts, respectively. These results indicate that our ANN can be used to predict pancreatic cancer risk with high discriminatory power and may provide a novel approach to identify patients at higher risk for pancreatic cancer who may benefit from more tailored screening and intervention.

## Introduction

Pancreatic cancer (PC) remains the fourth leading cause of cancer-related death in both men and women in the United States (Klein et al., 2013; American Cancer Society, 2017) despite its low incidence rate (Pannala et al., 2009). In 2017, a total of 53,670 new PC cases (3.18% of all new cancer cases) and a total of 43,093 associated deaths (7.17% of all cancer deaths) were recorded in the United States (American Cancer Society, 2017). The age-adjusted cancer-related death rate is increasing for PC, and it is predicted that PC will become the second most common cause of cancer-related deaths by 2030(Klein et al., 2013; Boursi et al., 2017). PC has a high mortality rate in part because cancer-specific symptoms in most patients (>80%) occur only at an advanced stage (Pannala et al., 2009; Klein et al., 2013,; Boursi et al., 2017).

According to the 2017 American Cancer Society (ACS) statistics, the recent 5-years survival rate for all stages of PC is 8.5% (American Cancer Society, 2017). The 5-years survival rates for patients with early-stage diagnosis can be as high as 20% (Winter et al., 2006; Howlader, 2011; Klein et al., 2013). However, only a small portion of patients (<15%) have surgically resectable disease at the time of diagnosis (Pannala et al., 2009). Furthermore, identification of individuals at high risk for PC or with early-stage disease is difficult due to the lack of a reliable screening tools, the absence of sensitive and specific biomarkers, and the low prevalence (Pannala et al., 2009; Yu et al., 2016; Boursi et al., 2017).

Recently, numerous studies have been focused on early detection of PC through the identification and validation of promising biomarkers (Grønborg et al., 2004; Gold et al., 2010; Klein et al., 2013). Further, the ability to detect pre-cancerous changes in the pancreas among high-risk individuals via Doppler ultrasound (US), endoscopic ultrasound (EUS), magnetic resonance imaging (MRI), computed tomography (CT) scan, or positron emission tomography (PET) has also been demonstrated in several clinical studies (Canto et al., 2004, 2006; Poley et al., 2009; Verna et al., 2010; Klein et al., 2013). Pancreatic tumors as small as 0.5 cm can be identified with diagnostic imaging, such as CT, MRI, or EUS. However, despite the high sensitivity of these techniques (Klein et al., 2013; Boursi et al., 2017), it is not practical or economically feasible to perform widespread PC screening in the general population due to the relatively low incidence rate (Klein et al., 2013; Boursi et al., 2017). However, these techniques can be used more efficiently and cost-effectively if employed in a high-risk subset of the population. For example, screening protocols are applied in patients with germline mutations associated with PC and patients with familial PC (Boursi et al., 2017). However, only 10–20% of all PC cases can be attributed to familial PC (Boursi et al., 2017).

Various epidemiologic and clinical characteristics are associated with occurrence of PC, including family history of PC (Permuth-Wey and Egan, 2009), inherited genetic variation/influence (Lichtenstein et al., 2000; Klein et al., 2013), anthropometric variables [e.g., body mass index (BMI)] (Pannala et al., 2008; Arslan et al., 2010; Hart et al., 2011; Klein et al., 2013; Association, 2014), lifestyle (e.g., smoking, drinking alcohol) (Iodice et al., 2008; Michaud et al., 2010; Lucenteforte et al., 2011; Klein et al., 2013), and medical comorbidities (e.g., pancreatitis, diabetes) (Lowenfels et al., 1993; Pannala et al., 2009; Ben et al., 2011; Klein et al., 2013; Boursi et al., 2017). New onset diabetes is considered one of the strongest predictors of PC, and numerous epidemiologic studies reported that the association between newly diagnosed PC and diabetes mellitus was ~50% (American Cancer Society, 2017; Boursi et al., 2017). Chari et al. (2005) showed that the 3-years cumulative incidence of PC among patients with new onset diabetes is 8 times higher than expected (Boursi et al., 2017). Hence, it can be stated that diabetes associated with PC may be a paraneoplastic phenomenon caused by the cancer (Pelaez-Luna et al., 2007; Sah et al., 2013; Boursi et al., 2017). Smoking also increases the risk of PC by a factor of two (American Cancer Society, 2017). Even the use of smokeless tobacco increases PC risk (American Cancer Society, 2017). Family history of PC is also considered a risk factor (American Cancer Society, 2017).

To our knowledge, no established screening strategy has been introduced for sporadic PC. The non-invasive precursor lesions known as pancreatic intraepithelial neoplasia (PanIN) progress from PanIN1 to PanIN3 and into PC within an undefined timeline (Hruban et al., 2000; Pannala et al., 2009; Yu et al., 2016). Brat et al. (1998) reported the presence of PanINs 1.4–10 years before the appearance of PC clinically. In another study, 114 CT scans in 45 patients (done either at or before PC diagnosis) were reviewed to estimate the timeline for progression of PC (Pannala et al., 2009). Multiple studies indicate that the radiographic features of unresectability and the onset of symptoms of the cancer appeared simultaneously (Gangi et al., 2004; Pelaez-Luna et al., 2007; Pannala et al., 2009). Pannala et al. (2009) stated that PC remains resectable when asymptomatic and thus is unlikely to be detected. It is estimated that symptoms manifest about 6 months after PC becomes unresectable (Pannala et al., 2009). Therefore, identifying those at high risk yet asymptomatic is very important to find PC while it is still resectable.

The artificial neural network (ANN), which is based on the brain's neural structure (Rosenblatt, 1958), raised the interest of scientific community worldwide in the field of medicine due to its potential for diagnostic and prognostic applications (Smith et al., 1988; Salim, 2004; Kamruzzaman et al., 2010; Patil and Mudholkar, 2012). It has been used in heart disease (Kamruzzaman et al., 2010), predicting headache, pre-diagnosis of hypertension (Sumathi and Santhakumaran, 2011), kidney stone diseases (Kumar and Abhishek, 2012), classifying breast masses to identify breast cancer (Das and Bhattacharya, 2008; Pandey et al., 2012), dermatologist-level classification of skin diseases/cancer (Bakpo and Kabari, 2011; Esteva et al., 2017), prediction of skin cancer and blood cancer (Payandeh et al., 2009; Esteva et al., 2017; Roffman et al., 2018a), and diagnosis of PC (Sanoob et al., 2016). As an example of the workflow in these applications, classification of skin cancer was performed via a single convolutional neural network, which was trained with a dataset of 129,450 clinical images (Esteva et al., 2017). In another study, an ANN model was created to diagnose PC based on a data set of symptoms (Sanoob et al., 2016). A total sample of 120 patients (i.e., 90 training samples and 30 testing samples) with 11 possible symptoms and 3 outcomes were considered for this model (Sanoob et al., 2016). The authors claimed that the ANN model has advantages over typical strategies for disease diagnosis (Sanoob et al., 2016).

Roffman et al. (2018a) took a novel approach to predict non-melanoma skin cancer by using personal health data (e.g., gender, race, Hispanic ethnicity, hypertension, heart disease, exercise habits, history of stroke, etc.) commonly available in electronic medical record (EMR) systems. The area under the conventional receiver operating characteristic (ROC) curve was 0.81 and 0.81 for training and validation, respectively (Roffman et al., 2018a). This study suggests that the ANN can be a convenient and cost-effective method in evaluating cancer risk for individuals (Roffman et al., 2018a). Likewise, the goal of this study is to develop an ANN to calculate risk for PC in the general population and to identify a high-risk population in a cost-effective manner by utilizing easily available personal health data.

## Materials and Methods

### Two Data Sources

The National Health Interview Survey (NHIS) (Blewett et al., 2017) was established in 1957 to monitor the overall health status of the United States through personal household interviews on a broad range of health topics. Numerous epidemiologic studies have been conducted using NHIS (Blewett et al., 2017; Roffman et al., 2018a). The NHIS datasets of 1997 to 2017 (Blewett et al., 2017) were used in this study. The target study population consisted of people with onset of pancreatic cancer <4 years prior to the survey date. Considering the time dependency of input features to the model, this 4-years cutoff on the pancreatic cancer group was selected after careful testing of different cutoffs on model performance to strike a balance between sample size and the predictive power of our model. After applying this cutoff, we have 645,217 respondents, 131 of whom had PC.

The Prostate, Lung, Colorectal, and Ovarian (PLCO) trial (NCI, 2018) is a randomized, controlled trial investigating whether certain screening exams reduce mortality from prostate, lung, colorectal and ovarian cancer. Between November 1993 and July 2001, 154,897 participants were enrolled, 767 of whom developed PC during 13 years of follow up. For this study, PC status, personal health data, family history, socio-behavior, lifestyle and dietary data have been extracted from PLCO datasets via an in-house Matlab code.

### Primary Outcome

The primary outcome of interest includes (1) the accuracy of model prediction for PC; and (2) the feasibility of individualized cancer risk stratification for tailored intervention.

### Predictors

A total of 18 personal health features were selected for use in the ANN for PC risk prediction based on literature review, biological plausibility, and clinical judgment. The details of these personal health features are given in Table 1. Some features are converted to binary format [one-hot encoding (Harris and Harris, 2014)] and the others are rescaled to fall between 0 and 1 (Roffman et al., 2018a). All these features were available in the NHIS dataset and most of them were also in the PLCO dataset.

**Table 1:** Description of the personal health features from NHIS and PLCO datasets used in the ANN.

**Variable**	**NHIS**				**PLCO**			
	**Cancer**		**No cancer**		**Cancer**		**No cancer**	
	**Mean (±SD)**	**% Missing**	**Mean (±SD)**	**% Missing**	**Mean (±SD)**	**% Missing**	**Mean (±SD)**	**% Missing**
**CONTINUOUS VARIABLES**								
Age	59.8 (17.1)	0.0	47.8 (18.1)	0.0	71.1 (6.3)	0.0	73.6 (5.9)	0.0
Diabetes age	55.2 (15.2)	1.8	48.4 (16.9)	2.3	N/A	100	N/A	100
Smoking age	19.3 (9.8)	0.9	18.6 (7.3)	1.6	18.8 (5.3)	0.7	18.6 (5.1)	0.6
Years quit	19.2 (16.6)	1.4	17.3 (14.2)	1.1	25.6 (13.6)	2.5	31.2 (12.5)	1.9
Pack-years	27.3 (22.9)	71.0	19.1 (20.5)	53.7	44.3 (33.6)	2.5	35.9 (29.3)	2.3
Vigorous exercise	44.1 (191.9)	0.0	89.2 (250.0)	4.4	N/A	100	N/A	100
Moderate exercise	85.2 (192.2)	1.0	118.6 (297.7)	5.5	N/A	100	N/A	100
Drinking frequency	78.5 (118.7)	0.0	77.1 (102.7)	1.3	N/A	100	N/A	100
Drinking amount	2.2 (3.9)	29.8	2.6 (2.8)	12.4	N/A	100	N/A	100
Binging frequency	10.7 (49.5)	30.6	13.7 (46.0)	13.3	N/A	100	N/A	100
Family members with PC	0.4 (1.4)	85.8	0.1 (0.6)	86.6	0.5 (2.3)	5.9	0.3 (1.8)	3.9
Family members >50 with PC	0.0 (0.0)	58.8	0.0 (0.3)	86.6	1.0 (0.0)	0.0	1.0 (0.0)	0.0
BMI	25.3 (5.3)	1.9	27.3 (6.0)	3.9	27.1 (4.5)	6.0	27.3 (4.9)	4.7
**DISCRETE VARIABLES**								
Male	48.8%	0.0	55.9%	0.0	42.6%	0.0	50.5%	0.0
Emphysema	7.6%	0.0	1.8%	0.1	3.2%	5.4	2.5%	3.8
Asthma	15.2%	0.0	11.4%	0.1	N/A	100	N/A	100
Stroke	6.6%	0.0	3.0%	0.1	2.6%	5.7	2.4%	3.8
Coronary heart disease	11.4%	0.5	4.6%	0.2	12.2	5.6	9.1	3.8
Angina pectoris	7.6%	0.0	2.5%	0.2	N/A	100	N/A	100
Heart attack	8.5%	0.0	3.6%	0.1	12.2%	5.6	9.1%	3.8
Other heart disease	16.6%	0.0	7.9%	0.2	N/A	100	N/A	100
Ulcer	20.9%	0.0	7.7%	0.2	N/A	100	N/A	100
Drink	75.8%	0.0	77.4%	1.2	N/A	100	N/A	100
Other cancer	9.0%	0.0	0.0%	0.0	2.2%	0.0	0.1%	0.0
Hypertension	52.6%	0.0	29.6%	0.0	41.9%	5.3	36.7%	3.8
Hispanic	7.6%	0.0	16.3%	0.0	2.2%	9.0	2.1%	5.7
**Diabetes**		0.0		0.1		5.5		3.9
Diabetic	26.1%		8.6%		12.7%		7.7%	
Prediabetic	0.0%		1.4%		N/A		N/A	
Not diabetic	73.9%		90.0%		87.3%		92.3%	
**Smoking**		0.0		0.8		0.0		0.0
Current	14.7%		20.3%		16.4%		10.3%	
Former	36.0%		22.2%		41.2%		41.7%	
Never	49.3%		57.5		42.4%		48.0%	
**Smoking frequency**		0		1.9		0.0		0.0
Every day	24.3%		37.5%		28.5%		19.9%	
Some day	4.7%		10.1%		N/A		N/A	
Quit	71.0		52.3%		71.5%		80.0%	
**Race**		0.0		0.0		0.0		0.0
White	78.2%		74.6%		82.8%		85.6%	
Black	14.2%		14.4%		4.7%		5.0%	
AINA	0.5%		0.9%		0.1%		0.3%	
Asian Indian	1.0%		0.9%		5.1%		3.6%	
Chinese	1.0%		1.0%		5.1%		3.6%	
Filipino	1.0%		1.0%		5.1%		3.6%	
Other	3.8%		6.9%		0.4%		0.5%	
Multiracial	0.5%		0.3%		N/A		N/A	

### Sample Size Considerations

All the data in the NHIS dataset from 1997 to 2017 and PLCO dataset were used to maximize the power and generalizability of the results. To investigate the performance of ANN on different datasets, three datasets were built:

1. DS1 = NHIS dataset (645,217 participants, with 131 PC cases)
2. DS2 = PLCO dataset (154,897 participants with 767 PC cases)
3. DS3 = NHIS dataset + PLCO dataset (800,114 participants with 898 PC cases)

After constructing and randomizing these three datasets, we used a train/validate/test scheme. The ANN was trained on 70% (training dataset) of the data using 10-fold cross-validation, while the remaining 30% was withheld for further testing (testing dataset). Cancer risk, sensitivity, and specificity were calculated for both training and testing datasets.

### Missing Data

Some entries for some respondents were missing because they did not respond, or the question was not applicable. The details of these missing data are given in Table 1. To address these missing data, we used the idea of one-hot encoding (Harris and Harris, 2014). Essentially, for each feature we create a binary variable indicating whether a respondent has a value for that feature. Then the missing value is set to −1, outside of the range of the “real” data.

### Statistical Analysis

Given the binary outcome, we developed our prediction model using the logistic activation function. The model was developed, and all analyses were performed using an in-house Matlab code.

### Artificial Neural Network (ANN)

**Figure 1:**
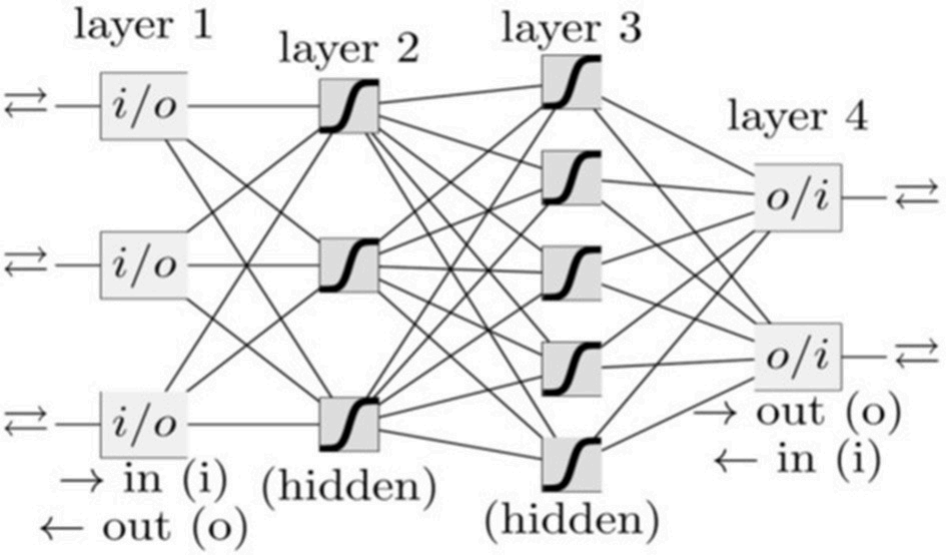
A schematic of an ANN. Each box and line represents a neuron and a weight, respectively. The number of weights grows rapidly with the number of neurons.

In our group, besides PC, we have also investigated a variety of other cancer types, such as lung cancer (Hart et al., 2018), prostate cancer (Roffman et al., 2018b), endometrial cancer (Hart et al., 2019), and colorectal cancer (Nartowt et al., 2019a,b) using ANN, Support Vector Machine, Decision Tree, Naive Bayes, Linear Discriminant Analysis, and Logistic Regression. Our results indicated that in general, ANN achieves the best performance as compared to other algorithms in terms of sensitivity, specificity, and AUC. Therefore, we used ANN in the present work. A schematic of an ANN model is shown in Figure 1. Our ANN had, in addition to the input and output layers, two hidden layers (each consisting of 12 neurons). The input features (between 0 to 1) and output (0 or 1) were split into 70/30 for training and testing datasets while keeping the ratio of the number of cancer cases to non-cancer cases constant. Within the training dataset, 10-fold stratified cross validation was used to evaluate the performance of models trained on the different datasets. Once the best model was chosen, we trained it on the full training dataset and then evaluated it on the test dataset. We used a logistic activation function and the sum of squared errors cost function. We trained our model using the standard backpropagation algorithm with simple gradient descent (http://ufldl.stanford.edu/tutorial/supervised/MultiLayerNeuralNetworks/), except that we used momentum to speed up the convergence. We batch trained our model (using the whole dataset at once) instead of online training (Roffman et al., 2018a). We ran the training for 5,000 iterations. The output of the ANN is a fractional number between 0 and 1. A higher output value means higher risk of PC. This fractional value can be transformed into cancer status (Yes or No) by choosing a threshold value above which the ANN will give a positive prediction for the cancer status (YES) or otherwise a “NO” for non-cancer. A variety of threshold values are tested to compute sensitivity and specificity after completion of the training. The selected threshold value from the training dataset is used to compute the sensitivity and specificity for the testing set.

### Model Performance Evaluation

The models trained on different datasets were evaluated based on the mean of the performance on the validation datasets. Specifically, we used the area under the ROC curve (AUC) as the measure of performance. This was chosen because in order to stratify the population into risk groups we want to have good discrimination (Metz, 1978).

Once the best model was selected, its performance on both the training and testing datasets was evaluated, testing the ability of the risk score to differentiate between the individuals with onset of PC and non-PC individuals. In addition to the AUC, the agreement between the predicted probabilities from the model and the observed outcomes are reflected from the training of the model.

### Risk Stratification

A risk stratification scheme was tested to demonstrate the potential application of our ANN model in the clinic. The scheme was designed to divide the population into three categories: low, medium, and high risk. These boundaries were conservatively selected using the training dataset, such that no more than 1% of respondents without cancer and with cancer would be categorized as high and low risk, respectively. However, the medium-high risk boundary could be selected to stratify more respondents with cancer in the high-risk category in case of low cost and/or potential harms in screening non-cancerous respondents. With these boundaries selected from the training data, the stratification scheme is then applied to the testing dataset to demonstrate the potential clinical application of the model. Per this risk stratification scheme, high-risk individuals could be screened immediately. The medium-risk and low-risk individuals could receive their standard regular and less frequent screenings, respectively.

## Results

### Model Selection

**Figure 2:**
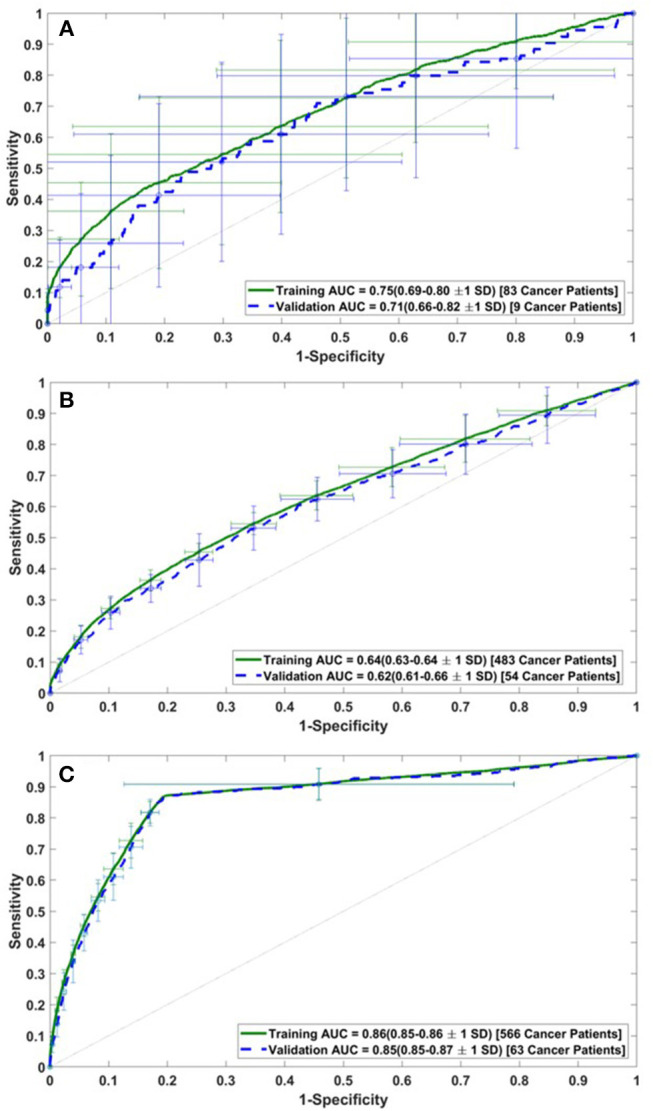
Receiver operating characteristic (ROC) plots for the training and validation datasets of **(A)** DS1, **(B)** DS2, and **(C)** DS3.

The performance of the model was assessed by calculating the AUC of the ROC plots for all three datasets (i.e., DS1, DS2, and DS3). For DS1, the AUC of the ROC plot is 0.75 ± 0.06 for the training sets, and 0.71 ± 0.11 for the testing sets (Figure 2A), while for DS2, these values are 0.64 ± 0.01 for training and 0.62 ± 0.04 for testing (Figure 2B). Similarly, the AUCs for DS3 are 0.86 ± 0.01 and 0.85 ± 0.02 for the training and testing sets, respectively (see Figure 2C). The best performance of the model was observed for DS3.

### Final Model Performance

**Figure 3:**
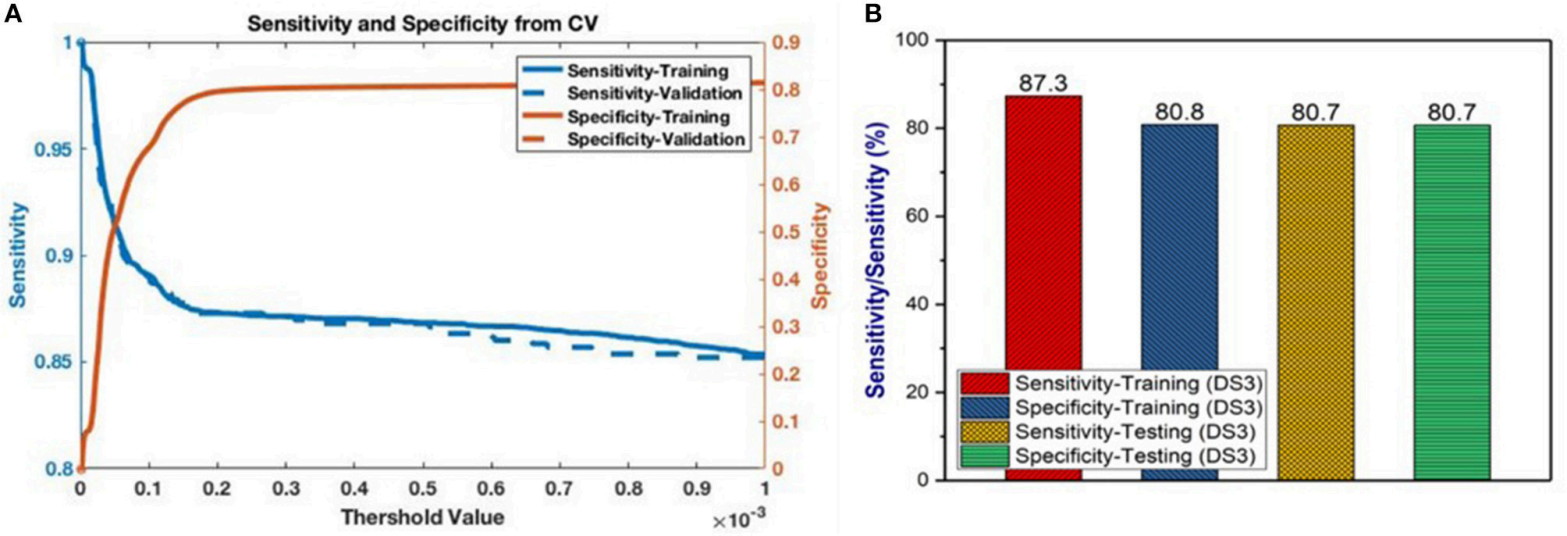
**(A)** Sensitivity and specificity from final training as functions of the cutoff values for DS3, and **(B)** representation of sensitivity and specificity values for DS3.

**Figure 4:**
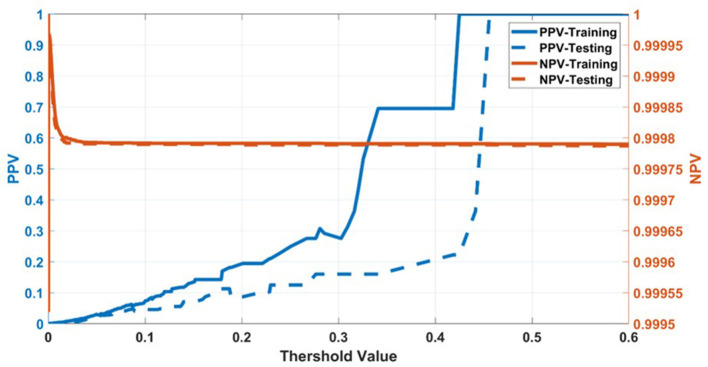
Positive predictive value (PPV) and negative predictive value (NPV) for training dataset of DS3.

Having selected the DS3 model, we train it on the full training dataset and evaluated it on the testing dataset. The sensitivity and specificity for both training and testing are plotted as functions of the threshold risk to study their trends (Figure 3A). Selecting the threshold risk that maximizes the sum of the sensitivity and specificity, we get specific values plotted in Figure 3B. The positive predictive value (PPV) and negative predictive value (NPV) are plotted as a function of threshold value shown in Figure 4. For the presented values of sensitivity and specificity of the DS3 training dataset, PPV and NPV values are 0.1% (95% confidence interval (CI): 0.09–0.100%) and 99.997% (95% CI: 99.996–99.997%), respectively. Similarly, for the DS3 testing dataset, 0.089% (95% CI: 0.084–0.095%) and 99.995% (95% CI: 99.993–99.996%) are PPV and NPV values, respectively for the presented values of sensitivity and specificity.

### Risk Stratification

**Figure 5:**
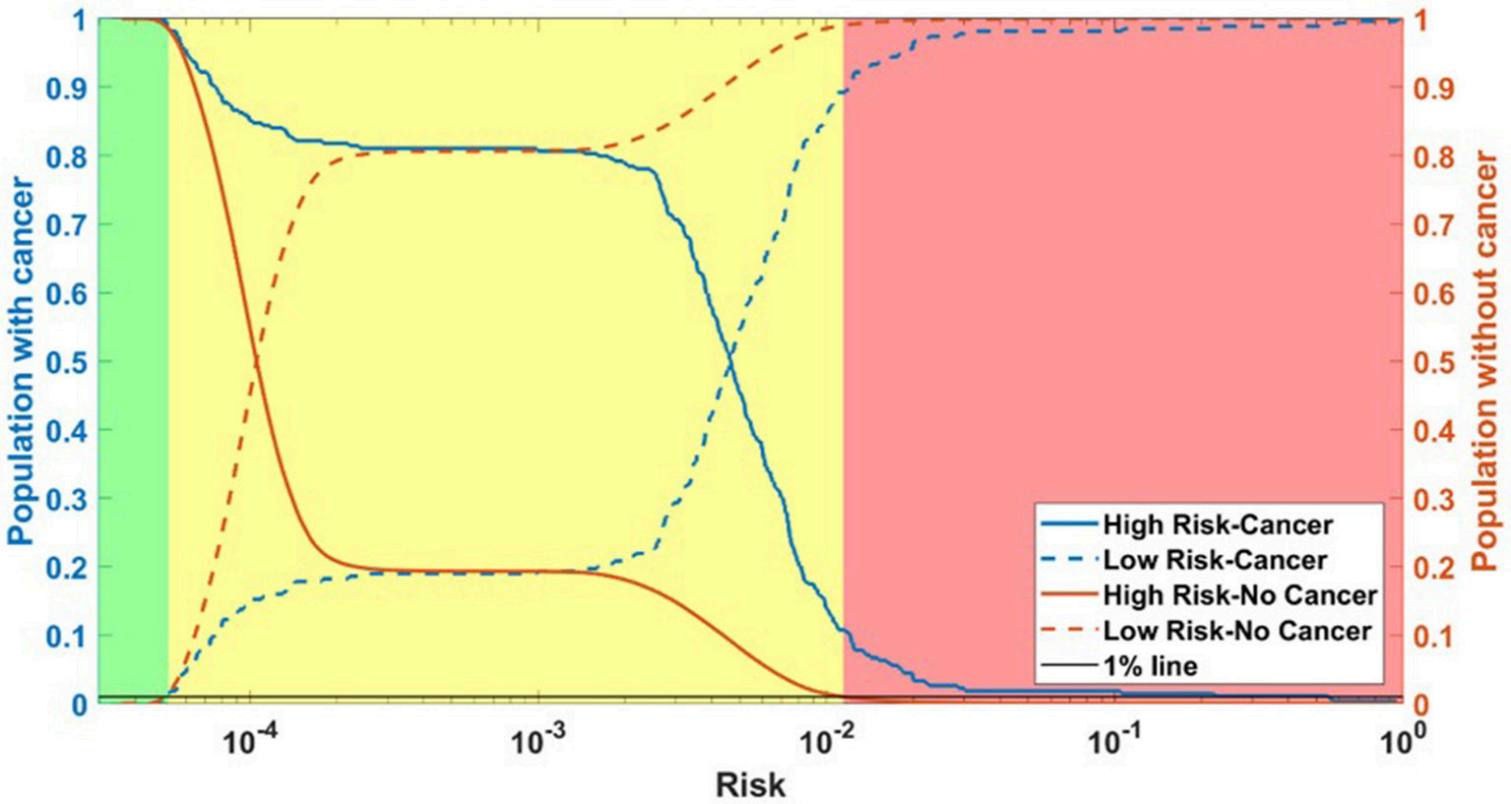
Population with Cancer (blue) and without cancer (orange) as functions of high-risk (solid) and low-risk (dashed). Assuming a 1% miss classification rate in the low- and high-risk categories (black line), individuals can be divided into 3 categories according to their cancer risk: high (red), medium (yellow), and low (green) for DS3.

Running through the DS3 dataset, the outputs of the ANN were categorized as low-, medium- and high-risk. The categorized fraction of the respondents with and without PC varied at different risk levels. It was clear from Figure 5 that most of non-cancer respondents were categorized in either low or medium risk while most of the respondents with cancers were either categorized as medium or high-risk. Risk stratification results for the testing datasets were summarized in Table 2.

**Table 2:** Risk Stratification for DS3 (NHIS and PLCO datasets combined).

**Data**		**# Low-risk (%)**	**# Medium-risk (%)**	**# High-risk (%)**
Training	PC	7 (1.1)	525 (16.5)	97 (15.4)
	Non-PC	8,490 (1.5)	545,368 (97.5)	5,594 (1.0)
Testing	PC	4 (1.5)	236 (87.7)	29 (10.8)
	Non-PC	3,717 (1.6)	233,653 (97.5)	2,394 (1.0)

## Discussions

In this study, risk of PC is predicted and stratified based on basic personal health data (NHIS and PLCO datasets) using a multi-parameterized ANN model. The model performance was evaluated by training and testing it on different datasets to determine its optimum performance. The best performance of the model was observed for DS3 with an AUC of 0.86 [CI 0.85–0.86 ±1 standard deviation (SD)] and 0.85 (CI 0.85–0.87 ±1 SD) for training and testing, respectively (Figure 2C). The best observed values for sensitivity and specificity for the training (testing) datasets of DS3 are 87.3% (80.7%) and 80.8% (80.7%), respectively. In 2017, the number of new cases of PC was 12.6 per 100,000 men and women per year (American Cancer Society, 2017). With our NPV value from the testing dataset being 99.995%, when our model predicts someone does not have cancer it is only wrong 0.005% of the time (5 per 100,000). For the DS3 testing dataset our PPV value is 0.09% (90 per 100,000). The group our ANN flags as having cancer is enriched more than 7-fold over the general population.

Because of the low number of PC cases for NHIS datasets (DS1), the model overfit and did not perform very well which is evident from the standard deviation in the validation AUC. The model also did not perform well for DS2 because the PLCO data consists of an enriched population of high-risk individuals with a higher median age. Also, there were a number of input features (e.g., alcohol use) that were completely absent in the PLCO datasets. Therefore, the model lost diversity and predictive power and relatively lower AUC values were observed. By combining NHIS with PLCO datasets, AUC value increased to 0.85, indicating a significant improvement in the discriminatory power of the model.

Currently, contrast-enhanced US, EUS, MRI, CT, and PET are the most promising modalities for PC screening (Verna et al., 2010; Klein et al., 2013). Each of these techniques has its advantages and limitations in screening for PC, but these techniques are often applied after the appearance of symptoms, which may be fatally too late in most cases. However, our ANN is focused on the early prediction and stratification of PC risk before symptoms appear. The results show that without any screening tests, the ANN produced very good predictions for PC. By comparing our results with already established screening modalities (i.e., EUS and MRI), PC risk was estimated with a high sensitivity and decent specificity. We stress that only personal health data (the type that is readily available in the EMR system) was used to reach this level of sensitivity and specificity.

The ANN can also be used to categorize the general public into low, medium, or high risk for PC based on easily obtainable personal health data in NHIS format. Reliable identification of high-risk patients who may benefit from tailored screening may improve a probability to detect PC at early stages. According to our testing results for the model, only 3 (1.9%) of respondents with cancer are incorrectly classified as low-risk, while only 2,394 (1%) of respondents in the total stratified population without cancer are false-positively categorized as high-risk (Table 2). With an AUC of 0.85, our model can effectively discriminate between respondents with and without PC (Figure 2).

Recently, a clinical prediction model has been used to assess PC risk with pre-diabetic and new onset diabetic patients (Boursi et al., 2017, 2018). For pre-diabetic study, a total number of 138,232 patients with new onset impaired fasting glucose (IFG) were selected where 245 individuals were diagnosed with pancreatic ductal adenocarcinoma within 3 years of IFG diagnosis. The prediction model included age, BMI, PPIs, total cholesterol, LDL, ALT and alkaline phosphatase. The reported AUC of the model was 0.71 (95% CI 0.67–0.75) (Boursi et al., 2018). By analyzing 109,385 onset diabetic patients including 390 PC cases, their model produced AUC of 0.82 (95% CI, 0.75–0.89) (Boursi et al., 2017). However, a comprehensive list of PC risk factors (54 in total) were used, e.g., age, BMI, change in BMI, smoking, use of proton pump inhibitors, and anti-diabetic medications, as well as levels of hemoglobin A1C, cholesterol, hemoglobin, creatinine, and alkaline phosphatase. This set of data requires specialized equipment to collect and may not be reportable by all members of the general public. In contrast, our ANN works on personal health data that are easily reportable by the general public while maintaining an AUC of 0.85.

Cai et al. (2011) developed a PC risk stratification prediction rule by studying 138 patients with chronic pancreatitis. A scoring method based logistic regression was used to develop the prediction rule. Hsieh et al. (2018) predicted PC in the patients with type 2 diabetes using logistic regression and artificial neural network models. In another study, Wang et al. (2007) predicted familial PC risk through a Mendelian model (i.e., PancPRO) that was built by extending the Bayesian modeling framework. The AUCs achieved by these models were 0.72 (Cai et al., 2011), 0.73 (Hsieh et al., 2018), and 0.75 (Wang et al., 2007), respectively. With lower AUCs as compared to the current study and being designed for specific conditions, these studies may not be widely used for the general public. In another study, a weighted Bayesian network was used for prediction of PC by combining PubMed knowledge and electronic health record (EHR) data (Zhao and Weng, 2011). A total of 20 common risk factors (i.e., age, gender, smoking, and/or alcohol use, weight loss, vomiting, nausea, fatigue, appetite loss, jaundice, abdominal pain, diabetes, depression, AST, ALT, albumin, alkaline phosphatase, GGT, glucose, bilirubin, CEA, and CA 19-9) associated with PC were used with PubMed knowledge to weigh the risk factors. Their network produced an AUC of 0.91 (95% CI, 0.869–0.951). Although these results are promising, the weighting has been calculated separately for each risk factor. If more risk factors are added, the prediction results will be different due to added weightings from PubMed knowledge. Secondly, in these studies, most features are clinical and hence not readily available. Our ANN's weights were fit on the training dataset and if more risk factors are added, updating the weights to include the new factors can be done quickly by re-fitting the ANN.

Nakatochi et al. (2018) presented a PC risk prediction model in the general population in Japan with AUC of 0.63. However, their model was based on data including directly determined or imputed single nucleotide polymorphisms (SNPs) genotypes. While our ANN model performed considerably well to predict PC on the basis of commonly available data in the EMR, inclusion of personal high-risk features for PC (e.g., pancreatic cysts, family history etc.) could potentially improve the performance of the model. Our approach is also distinct from previous studies because it is based on survey data representative of the general population. The previous studies are based on either one or more clinical conditions or smaller sample sizes. Furthermore, the developed ANN may be very helpful to primary care physicians due to its ability to stratify people into various risk categories. Higher risk people could be referred to a diagnostic department for more tailored and intensive assessments. We envisage that this model can be integrated into an EMR system or be available on websites and portable devices, such as mobile phones and tablets. This will be very helpful for the clinicians to calculate the PC risk of their patients immediately after entering their data. More importantly, with the tool embedded in the clinical workflow, pancreatic cancer could be detected at an early stage, hence improving the survival rate in the long run.

## Conclusion

We reported an ANN that can be used to predict pancreatic cancer with a sensitivity of 80.7%, a specificity of 80.7%, and an AUC of 0.85 based solely on personal health data. In addition, the developed ANN was able to stratify people into low, medium and high cancer risk for more tailored screening and risk management. Compared to current screening techniques, this ANN is non-invasive, cost-effective, and easy to implement with readily available personal health data. More data and testing would be needed to further improve the performance of the ANN in order to facilitate its application in the clinic.

## Author Contributions

WM analyzed data, produced results, and wrote technical details. GH provided first version of working code. GH, BN, JF, KJ, and YL provided consultation, produced technical details and reviewed the manuscript. JD generated research ideas and reviewed manuscript.

### Conflict of Interest Statement

The authors declare that the research was conducted in the absence of any commercial or financial relationships that could be construed as a potential conflict of interest.
